# Nurses' perceived barriers to the implementation of a Fall Prevention Clinical Practice Guideline in Singapore hospitals

**DOI:** 10.1186/1472-6963-8-105

**Published:** 2008-05-18

**Authors:** Serena SL Koh, Elizabeth Manias, Alison M Hutchinson, Susan Donath, Linda Johnston

**Affiliations:** 1 School of Nursing, Faculty of Medicine, Dentistry and Health Sciences, University of Melbourne, Australia; 2 Clincial Epidemiology and Biostatistics Unit, Murdoch Children's Research Institute, Melbourne, Australia; 3 Department of Paediatrics, Royal Children's Hospital, Melbourne, Australia; 4 Neonatal Nursing Research, The Royal Children's Hospital and Murdoch Children's Research Institute, Melbourne, Australia

## Abstract

**Background:**

Theories of behavior change indicate that an analysis of barriers to change is helpful when trying to influence professional practice. The aim of this study was to assess the perceived barriers to practice change by eliciting nurses' opinions with regard to barriers to, and facilitators of, implementation of a Fall Prevention clinical practice guideline in five acute care hospitals in Singapore.

**Methods:**

Nurses were surveyed to identify their perceptions regarding barriers to implementation of clinical practice guidelines in their practice setting. The validated questionnaire, 'Barriers and facilitators assessment instrument', was administered to nurses (n = 1830) working in the medical, surgical, geriatric units, at five acute care hospitals in Singapore.

**Results:**

An 80.2% response rate was achieved. The greatest barriers to implementation of clinical practice guidelines reported included: knowledge and motivation, availability of support staff, access to facilities, health status of patients, and, education of staff and patients.

**Conclusion:**

Numerous barriers to the use of the Fall Prevention Clinical Practice Guideline have been identified. This study has laid the foundation for further research into implementation of clinical practice guidelines in Singapore by identifying barriers to change in acute care settings.

## Background

Patient care and outcomes could be significantly improved if knowledge gained from health research is better translated into practice [1]. The need to demonstrate clinically effective care is widely acknowledged yet research knowledge has been slow to influence practice. Barriers to implementing research in healthcare exist at many levels including the individual practitioner, the clinical team, the practice setting and the organizational context [2]. A plethora of literature has explored the research-practice gap phenomenon. The average practitioner, nurse or clinician does not have the time or the skills to locate and appraise the large volume of research publications. Furthermore, nurses may be uncertain about which treatment options to use when research results or advice from experts are conflicting [3]. These sentiments have been shared and confirmed by numerous studies which have consistently highlighted the barriers to research utilisation as time constraints, lack of awareness of available research literature, insufficient authority to change practice, inadequate skills in critical appraisal and lack of support for implementation of research findings [2,4-7]. In response to these findings, synthesis of published research studies and the development of clinical practice guidelines have emerged as tools to facilitate the application of research evidence. Guidelines serve as an important vehicle for translating thcomplex research findings into recommendations; overcoming barriers to research use such as the requirement for skills in critical appraisal and synthesis; and removing the requirement for interpretation of large volumes of scientific studies [8].

The Institute of Medicine describes clinical practice guidelines (CPGs) as 'systematically developed statements to assist practitioner and patient decisions about health care for specific clinical circumstances' [9]. The successful implementation of CPGs reduces inappropriate variation in practice and provides a set of instructions for clinical decision-making to improve patient safety, improve patient outcomes, and promote cost effective and quality care [10-14]. Despite wide promulgation, guidelines have had limited effect on changing professional behavior and practice [15-18]. Most studies of CPG implementation strategies have focused on changing the performance of doctors, and a minority have targeted nurses or other health professionals [19,20]. The findings of extensive research, from numerous studies, concur that passive dissemination of guidelines alone is usually insufficient to change clinical behavior and practice [11,12,21], while other interventions have small to moderate effects at best [22]. There is 'no magic bullet' [23] and little evidence exists to indicate which interventions to use for specific problems and settings [22,24]. Grimshaw et al [19] in their latest systematic review argued that across all combinations of interventions, multifaceted interventions did not appear to be more effective than single interventions. Nonetheless, they concluded by highlighting that it is plausible that multifaceted interventions built upon a careful assessment of barriers to implementation of guidelines may be more effective than single interventions under different circumstances. However, there is still insufficient rigorous evaluative research on implementation strategies for clinical practice guidelines in hospitals.

Theories of behavior change suggest an analysis and understanding of factors that prevent or motivate change in behavior might be helpful in tailoring guideline implementation strategies [25-30]. There has been widespread acknowledgement that tailoring implementation strategies require creativity in addition to an understanding of the key barriers to change [25,30]. However, there is a lack of empirical evidence to support any particular theory or framework for guiding development of strategies to influence change in nursing practice and there is no clear-cut basis for suggesting which specific interventions for which barriers to change are most effective. According to the NHS Centre for Reviews and Dissemination [31], implementation strategies need to be tailored to the local context; no single approach will have universal applicability. This message is reinforced by the development of the Promoting Action on Research Implementation in Health Services (PARIHS) framework [32-37], which proposed that the most successful implementation occurs when evidence is robust; the context is receptive to change with sympathetic cultures, strong leadership and appropriate monitoring and feedback systems; and when there is appropriate facilitation of change [32]. However, the effectiveness and utility of this framework in facilitating guideline implementation requires validation by further empirical research.

Grol [26] and Grol and Grimshaw [38] suggest that, in designing an effective implementation strategy, it is necessary to target local needs, barriers to and facilitators of, behavior change. These may include both structural and attitudinal factors and appropriate interventions might be targeted at both the structure and the process of care. Numerous studies identified that potential barriers to, and facilitators of, change can act at six different levels: 1) the innovation (feasibility, accessibility); 2) individual professional (knowledge, skills, attitudes, habits); 3) patient (knowledge, compliance); 4) social context (colleagues, authorities); 5) organizational context (available resources, organizational climate, structures); and 6) economic and political context (financial arrangements, regulation, policies) [2,39,40]. These factors underpin health professionals adoption of, and adherence to, the protocols developed from the clinical guidelines.

Recent work on efforts to implement fall prevention guidelines have incorporated locally targeted multi-intervention approaches in their fall prevention programme [41-43]. In response to the failure of two published, model-based, fall guidelines implementation projects, it was learnt that understanding local barriers and employing a locally tailored multifaceted implementation strategy are fundamental to successful implementation. However, research on issues about implementation of guidelines on fall prevention is non-existent in Singapore.

The Ministry of Health, Singapore (MOH), Nursing CPG on 'Prevention of Falls in Hospitals and Long Term Care Institutions' (Table 1) has been developed by the MOH taskforce. Since 2003, MOH has employed a working committee to develop CPGs, which are then disseminated to the acute care hospitals in Singapore. However, appropriate mechanisms to promote transfer and uptake of knowledge to change practice are unclear because the MOH does not give guidance on how to implement the guidelines and promote their use. It is anticipated that the identification of nurses' perceptions about barriers to implementation of the Fall Prevention CPG in acute care hospitals in Singapore, will assist in the process of selecting and more appropriately targeting implementation strategies locally to change nursing practice and reduce the burden of falls. The incidence of falls in the acute care hospitals in Singapore were low, between 0.68 and 1.44 per 1000 patient days [44], compared to international studies conducted in the United States, United Kingdom and Australia, which reported the incidence of falls ranging from 2 to 12 per 1000 patient days [45-47]. Despite the comparatively low fall rates in Singapore, the fall-associated injury rate was high, between 24.4 and 71.7% compared to international fall-associated injury rates which ranged between 5 to 10% [48,49]. The current study results indicate a substantially lower rate of falls in hospitals in Singapore, with a conversely higher rate of injuries associated with falling. In a recent large-scale Taiwanese study, Chen et al. [50] pointed out that the rate of patient falls in the domestic setting averaged 0.03%, far lower than the average reported internationally (0.25%). However, the proportion of falls associated with injury is far higher in Asian countries than overseas [50]. The reason for this may lie in the culture of reporting in Asia. Anecdotally, many falls are unreported by patients and relatives when there is no associated injury because they deem the fall to have been uneventful.

**Table 1:** Summary of Recommendations of Nursing CPG on 'Prevention of Falls in Hospitals and Long Term Care Institutions'

**Assessment & Interventions**	**Recommendations**
Assessment of Fall Risk	All patients admitted to hospitals should undergo falls risk assessment at the point of admission, within 24 hours, to identify those at higher risk of falls.
	Risk assessment should be multi-dimensional and include medical, functional and behavioral assessments of patients. No one risk screening tool alone will identify all persons at risk or risk factors.
	In acute care settings, reassessment of fall risk should be carried out at least twice a week and when there is a change in patient's status or environment.

Risk Factors Contributing to Falls	A fall risk assessment should include the following:
	• Medical
	- History of falls
	- Medications associated with increased falls risk
	- Secondary or specific diagnoses known to affect fall risk
	- Postural hypotension
	- Seizures, dizziness, vertigo
	• Functional
	- Altered mental status
	- Altered elimination status
	- Impaired/deterioration of activities of daily living
	- Impaired mobility or gait
	- Poor visual acuity
	• Behavioral
	- Poor safety awareness
	- Lack of insight into own health condition
	- Risk taking behavior

Multifactorial Falls Prevention Approach	A falls prevention programme should comprise multifactorial interventions incorporating both general and individual-specific/tailored strategies:
	▪ Environment safety
	▪ Identification systems
	▪ Interventions for patients with altered mental status
	▪ Interventions for patients with altered elimination status
	▪ Mobility and exercise
	▪ Medication review
	▪ Education
	The fall prevention programme should involve all members of the multi-disciplinary healthcare team.

Post Fall Analysis and Management	All patients who experience an inpatient fall should undergo a post-fall assessment.
	The post-fall assessment should be accompanied by:
	▪ Attention to patients' injuries
	▪ Medical review to exclude acute causes of fall
	▪ Investigation into the circumstances of fall

The aim of the present study was to gain an understanding of perceived barriers to implementation of the Fall Prevention CPG in acute care hospitals in Singapore. This study was undertaken as part of a larger study, which aimed to develop a multifaceted implementation strategy targeting the perceived barriers, to effectively implement this guideline, to facilitate change in nursing practice, and to reduce the burden of falls in acute care hospitals in Singapore.

## Methods

### Design

A survey design was chosen to assess the perceived barriers to change by eliciting nurses' opinions with regards to barriers to implementation of clinical practice guidelines in their practice setting. This was undertaken with the administration of a questionnaire to nurses working in five acute care hospitals in Singapore.

### Setting

All the acute care general hospitals (n = 5) in Singapore were involved in this study. The hospitals were acute care tertiary teaching facilities with an average bed capacity of 1000. These five hospitals had similar characteristics in terms of the range of clinical services provided, types of patients seen, and bed capacity to nursing staff strength.

### Sample

All nursing staff (n = 1830) working in the medical, surgical and geriatric units (n = 66) in the study hospitals during the four-week survey distribution time frame were invited to complete the questionnaire. These areas were selected as they comprised the majority of clinical settings of the hospitals.

### Data collection

The validated questionnaire titled, 'Barriers and facilitators assessment instrument', developed by Peters, Harmsen, Laurant, & Wensing [51], and designed to elicit perceptions of the barriers to implementation of CPGs in practice, was administered. Administration of the questionnaire took place over a period of three months from September to November 2005 for the five participating hospitals. A total of 1830 questionnaires (250 at Hospital A; 320 at Hospital B; 360 at Hospital C; 450 at Hospital D; and 450 at Hospital E) were distributed. Questionnaires were placed in personally addressed envelopes along with an explanatory letter of invitation. To facilitate distribution, the envelopes were hand delivered by one of the researchers or a hospital research collaborator to the nurse managers of the participating wards, who then accepted responsibility for distribution to all nurses in their respective areas. Each questionnaire took approximately 15 minutes to complete. Respondents were requested to return the completed questionnaire by placing the envelope into the 'return' box that was provided in each ward. A four-week time frame was allowed from distribution to return date. In the week before the due date an email was sent to the nurse managers to remind all nursing staff of the approaching due date for questionnaire return.

### Instrument

The questionnaire was developed by Peters et al [51] and applied in guideline implementation studies in the Netherlands to measure barriers to and facilitators for improvement of patient care, with a special focus on identifying physicians' and nurses' perceived barriers to change. Psychometric qualities, in particular item response and range, had been measured by the developers of the instrument. The first part of the instrument consisted of rating various possible barriers to, and facilitators of, the general implementation of a 'directive or innovation'. The second part of instrument consisted of identification of barriers to, and facilitators of, implementation of a specific 'directive or innovation' in practice. As recommended by the authors of the tool, six questions were reworded to address the specific guideline being employed in the study, specifically, the words 'directive or innovation' in the original questionnaire were replaced with the words 'Fall Prevention CPG'. Six questions which were not relevant in the context of the implementation of the CPG in Singapore were removed from the instrument. A total of 21 items remained. This questionnaire comprised two sections. The first section contained the 21 randomly ordered items from the 'Barriers and facilitators assessment instrument' [51]. Respondents were required to rate, on a 5-point Likert-type scale, ranging from 'Fully agree', which corresponded to a score of five to 'Fully disagree', which corresponded to a score of one. The scores related to the extent to which they believed each item was a barrier to the implementation of the CPG in practice. Items were grouped into four categories: 1) innovation characteristics, 2) care provider characteristics, 3) patient characteristics, and 4) characteristics of the organizational, social, political and societal context. Section Two of the questionnaire included a series of demographic questions.

Prior to distribution, the questionnaire was pre-tested for understandability of the questionnaire wording had relevance in the Singapore health care context, and to attain estimates of the amount of time required to complete the questionnaire. It was administered to five nurse managers and five nurse clinicians who did not work in the hospital in which the study was being conducted. Based on respondents' recommendations, some minor rewording of the barrier and demographic questions were undertaken.

### Data analysis

Data were entered into, and analyzed, using SPSS version 11.0 (SPSS Inc, Chicago, Il, USA). Frequencies and descriptive statistics were employed to describe the demographic characteristics of respondents. Frequencies, described as percentages of responses, and descriptive data, reported as means, were used to analyze the questions from the 'Barriers and facilitators assessment instrument'.

### Ethics

The study was approved by the Human Research Ethics Advisory Group of The University of Melbourne and the Institutional Review Boards of the five participating hospitals. Completion of the 'Barriers and facilitators assessment instrument' was voluntary. Consent to participate was implied with the return of completed questionnaires, and the questionnaires were completed anonymously. To avoid coercion, following distribution of the questionnaires the nurses were invited to voluntarily complete the survey and place the completed anonymous questionnaire in the 'return' box at any time within the study time frame. Regardless of their decision about participation, the nurses were also assured that their employment status within the organization would in no way be affected.

## Results

### Demographics

A total of 1467 nurses returned the questionnaires (80.2% response rate). Table 2 shows the demographic characteristics of the respondents for the five hospitals in Singapore. The mean age of respondents was 29.5 years (range: 18 – 62 years), while the mean number of years working as a nurse was 7.6 years (range: 0.08 – 40 years). About 34% (502) of respondents had research experience. The demographic characteristics of the nurse respondents were consistent throughout the five acute care hospitals in Singapore.

**Table 2:** Sample Demographics (N = 1467)

**Variables**	**N (%)**	**Mean (SD)**
**Gender**		
Male	30 (2.0)	
Female	1437 (98.0)	
**Age (years)**		29.52 (8.57)
**Experience Nurse (years)**		7.57 (8.34)
**Practice Setting**		
Medical	853 (58.2)	
Surgical	366 (25.0)	
Geriatrics	103 (7.0)	
Mixed-discipline	144 (9.8)	
**Highest Qualification**		
Certificate in Nursing	300 (20.4)	
Diploma in Nursing	662 (45.1)	
Advanced Diploma in Nursing	109 (7.4)	
Degree in Nursing	364 (24.8)	
Masters in Nursing	7 (0.5)	
Others	25 (1.7)	
**Current Position**		
Enrolled Nurse	402 (27.4)	
Midwife	5 (0.3)	
Staff Nurse	781 (53.2)	
Senior Staff Nurse	197 (13.4)	
Nurse Manager	44 (3.0)	
Nurse Clinician	21 (1.4)	
Senior Nurse Manager	5 (0.3)	
Others	12 (0.8)	
**Research Experience**		
Yes	502 (34.2)	
No	965 (65.8)	
**Types of Research Experience**		
Undertaken research as part of Diploma/Advanced Diploma programme	320 (46.6)	
Undertaken research as part of Degree/Masters programme	153 (22.3)	
Undertaken research as a Principal Investigator	14 (2.0)	
Undertaken research as a Collaborator	27 (3.9)	
Data collection for someone else's research project	81 (11.8)	
Journal club participation	30 (4.4)	
Undertaken education/courses in research	61 (8.9)	

### Barriers to implementation of guidelines

The item characteristics perceived by nurses as barriers to implementation of CPGs in practice are summarized in Table 3. The barriers to implementation of guidelines were identified from the combined percentage of 'disagree' and 'fully disagree' responses to the positive questions and the combined percentage of 'agree' and 'fully agree' responses to the negative questions. From the 21 questions, 16 item characteristics/barriers were identified. Overall, analysis showed that the greatest barriers to implementation of CPGs reported in five acute care hospitals in Singapore were: 1) knowledge and motivation (82.4%; range: 77.3% – 90.8%); 2) availability of support staff (77.8%; range: 69.7% – 83.9%); 3) access to facilities (73.3%; range: 68.6% – 79.6%); 4) health status of patients (55.7%; range: 53.0% – 61.4%); and 5) education of staff (49.4%; range: 26.8% – 55.7%).

**Table 3:** Summary of Barriers (N = 1467)

	Hospital A (N = 198)	Hospital B (N = 250)	Hospital C (N = 296)	Hospital D (N = 375)	Hospital E (N = 348)	Mean (N = 1467)
	
**Barriers/Characteristics**	%	%	%	%	%	%
**INNOVATION CHARACTERISTICS**						
Compatibility	6.1	10.4	4.8	11.5	8.6	8.5
Time Investment	15.1	27.7	19.7	29.1	27.9	24.7
Specificity, flexibility	26.3	26.8	10.8	16.9	22.7	20.2
Didactic benefit	5.0	5.6	3.0	2.9	3.4	3.8
Attractiveness	8.6	3.6	6.1	8.8	6.9	6.9
						
**CARE PROVIDER CHARACTERISTICS**						
Attitude, role perception	21.2	21.2	17.9	17.3	19.9	19.2
Knowledge and motivation	79.8	90.8	78.7	77.3	86.5	82.4
Doubts about the innovation	10.6	15.6	7.8	15.7	15.5	13.6
Life style, working style	4.5	12.4	6.8	11.2	6.9	8.5
Education	26.8	55.2	55.7	46.9	55.1	49.4
Involvement	30.7	45.2	43.6	45.6	39.6	41.6
						
**PATIENT CHARACTERISTICS**						
Ethnicity	34.3	43.6	41.3	48.5	38.0	41.8
Health status	54.1	54.4	53.0	61.4	54.0	55.7
						
**CONTEXT CHARACTERISTICS**						
Group norms, socialisation	14.6	37.2	29.4	29.2	37.7	29.6
Leadership	3.0	3.6	3.4	7.2	4.6	4.4
Supporting staff	69.7	82.0	72.0	78.4	83.9	77.8
Facilities	69.2	79.6	68.6	75.5	72.4	73.3

These top five barriers identified by respondents were consistent throughout the hospitals. 'Knowledge and motivation', 'supporting staff', and 'facilities' were in the top three barriers nominated by nurses at each of the five hospitals. 'Education' was in the top five barriers nominated by nurses at four hospitals. At two hospitals, 'ethnicity' was in the top five barriers identified. The following categorizations had been identified from the original questionnaire. Two of the top five barriers identified; 'knowledge and motivation' and 'education of staff', were considered 'care-provider' characteristics. 'Availability of supporting staff' such as a lack of a fall nurse specialist or change champion, and 'access to facilities' such as lack of resources or equipment, for example bed alarms, were categorized as 'context' barriers, while, the factor 'health status of patients' was related to the 'patient' characteristics. Leadership (4.4%; range 3% – 7.2%), didactic benefit (3.8%; range: 2.9% – 5.6%) and attractiveness of the guidelines (6.9%; range: 3.6% – 8.8%); both considered 'innovation' characteristics, were not perceived as major barriers.

## Discussion

This study is the first to report an examination of the Singaporean context in which guideline implementation may be influenced by an understanding of the local barriers to change in acute care hospitals so as to assist in the process of selecting and more appropriately targeting implementation strategies locally. Advocates for an evidence-based approach to guideline implementation have advised that prior to choosing one or more interventions, decision-makers need an understanding of the target group and setting and potential facilitators of, and barriers to change [38]. The results of our study will inform the development of implementation strategies targeting the barriers to change, to facilitate the role of MOH in ensuring the effective implementation of CPGs and adoption of evidence-based practice.

The barriers most frequently mentioned by nurses in this study were related to care providers and the context in which practice occurred. Specifically, care provider characteristics, such as knowledge and motivation, were nominated as important barriers to implementation of guidelines by 82.4% of nurses, which is similar to the findings of other international studies [51,52]. The expanding body of research and the increasing emphasis on evidence-based practice make it difficult for any practitioner to be aware of, familiar with, and able to critically apply, every applicable guideline to practice [53,54]. This is especially so in the Singapore context where 19% of nurses hold a Bachelors or Masters university qualifications (Singapore Nursing Board, 2005). This concern is also highlighted in this study in which the majority of nurses (65%) are either certificate-trained in the technical institute or possess only a diploma in nursing from the polytechnic. These nurses were neither exposed to research nor evidence-based practice within their curriculum. Moreover, our results indicate that 66% of respondents had no research experience and even fewer (24%) had a degree qualification. These results are not surprising in the Singapore context, where nurses are starting to work towards attainment of degree qualifications.

A systematic review of the literature to identify barriers to guideline adherence yielded similar results to those found in the present study, identifying care provider characteristics, particularly knowledge, as a highly reported barrier in 38% of surveys [52]. Our findings are also similar to those of Peters et al. [51], in three different implementation studies in the Netherlands involving 190 general practitioners (GPs); 40 GPs; and 160 midwives respectively. Eighty percent reported knowledge and motivation as a perceived barrier to change.

Appropriate knowledge and attitudes are necessary, but not sufficient, for guideline adherence [55]. Practitioners may still encounter barriers to adoption of guideline recommendations due to the guideline itself, patient, or social/environmental factors [52]. Furthermore, adherence to guidelines may require changes that are beyond the control of the practitioner, such as acquisition of resources or facilities [56,57]. This is clearly a challenging area that hospitals need to explore given their tight budgets and restrictions to additional resources. Context factors, such as lack of equipment or facilities; insufficient staff; and lack of opinion leaders, described by respondents as barriers in the study by Cabana et al [52], were congruent with those context characteristics identified in our study. This study found a high percentage of respondents (73.3%) perceived a lack of facilities and equipment such as bed alarms to be a barrier to the implementation of guidelines.

Currently, in the Singapore context, the MOH guidelines are simply disseminated to nurses through distribution the hospital wards in conjunction with a single 'road-show' to inform them of the publication. Seventy-seven per cent of respondents reported 'availability of supporting staff' as a barrier to implementation of guidelines. These findings were consistent with international studies where perceptions of general practitioners similarly identified lack of supporting staff as a barrier to change [51,58] This finding clearly highlighted the critical need for appropriate facilitation which has been echoed by PARIHS [36]. The nurses also perceived that a change champion or a facilitator leading and supporting the change to implement the guidelines in their respective hospitals would result in change in their practice.

Leadership is vital in effective guideline implementation [28,32,35,37]. It is encouraging that few nurses in the present study (4.4%) perceived leadership as a barrier to implementation. It could be argued that this illustrates that the nurses believe that their current leadership provides strong support and direction when guidelines are implemented.

The inability to reconcile patient characteristics (health status and ethnicity) with guideline recommendations was also identified as a barrier to change and these results are similar to those of other studies. This particularly could be explained by the multi-racial context in Singapore where nurses find such cultural differences difficult when implementing certain recommendations in the guideline, such as lack of interpreters to inform patients about risk of falling.

It is encouraging to note that most respondents in our study did not rate any 'innovation' characteristics as barriers to the implementation of guidelines which suggests that the nurses believe in the innovation and evidence, and find the recommendations of the guideline to be compatible with their daily practice. These results were similar to the reported perceptions of midwives in the study by Peters et al[51] It is possible the reason nurses believed the characteristics of the innovation did not present barriers to implementation is that the Fall Prevention CPG was locally adapted by the MOH workgroup, making it relevant to, and compatible with, the Singapore context [51,52].

Change management theories are instructive when implementing strategies to facilitate change in practice. There is no single change theory that dominates behavioral change and many concepts in different theories overlap [38]. However, change theories are helpful in explaining behavior and nurses' propensity to change practice following the implementation of the CPG at acute care hospitals in Singapore.

The environmental context within which the nurses practice is a key determinant of guideline adoption to facilitate a change in practice. Behavioral theory based on conditioning and controlling behavior, emphasizes the importance of the environmental context of behavior, suggesting that environmental cues and reinforcements are central in encouraging and maintaining behaviour [59]. The main strategies used in the present study, consistent with the stimuli that Skinner described were: reviewing performance and providing feedback to care providers, giving reminders, providing incentives and instituting sanctions such as policies related to specific actions. Environmental and organizational supports, which enabled and reinforced the use of the CPG, operated at two levels in the acute care settings in Singapore. At the clinical level, strategies such as reminders, which integrated the CPG into the process of care, were found to provide important environmental support for change. Organizational supports for guideline implementation such as policy revision to comply with the Fall Prevention CPG and accreditation standards, were important stimuli for change in practice. It has been suggested that the provision of such environmental and organizational support can help to build positive attitudes among care providers, which strengthens the drive for change [60].

Social influence theory emphasizes the role of others in decision making about behavior, postulating that factors such as customs, habits, beliefs of peers and prevailing practices and social norms shape the change of practice [61]. Social influence theory can help to expand our understanding of the social processes which influence success of guideline implementation. Strategies to promote behavior change through social influence, including the use of change champions [62] and educational sessions [63] have been shown to be effective and were used in the present study. The opinions of peers and change champions play a major part in influencing the attitudes of care providers and, most importantly, their decisions to change practice [64-66]. The value of this approach lies in its emphasis on professional communication, whereby care providers constantly look to each other for support, approval, role models, information, and feedback. Collectively, these theories were useful in guiding development of the multifaceted intervention to target barriers to change, promote clinical behavior change, and ultimately improve patient care and outcomes.

Guided by theories of practice change [25-29], the implementation strategy (Table 4) targeted perceived barriers to implementation of guidelines identified in the present study. Interventions included conduct of educational sessions; facilitation and support by change champions including a fall nurse specialist; allocation of resources and equipment; revision of the hospital fall prevention policy; reminders and identification systems; and audit and feedback.

**Table 4:** Interventions in implementation strategy

**Barriers**	**Strategy**	**Targeted Interventions**
Lack of supporting staff	Change champions	An implementation team, comprising the Deputy Director of Nursing, seven Nursing Unit Managers, four senior nursing staff and a geriatric physician, oversaw the project in terms of planning and implementing the interventions with the research team.
		Senior nursing staff were engaged as change champions to reinforce and encourage nurses to adhere to the strategies recommended in the guidelines.
		A 'fall nurse specialist' was employed to prescribe interventions according to risk factors identified; monitor compliance of nurses with the interventions; educate high-risk patients, their families and carers on fall prevention interventions; and conduct post-fall assessments and evaluations.

Lack of knowledge and education	Educational sessions	Educational sessions were aimed at promoting and supporting the adoption of the recommendations in the fall prevention guidelines. These interactive workshops included discussion of the importance of fall prevention, the role of fall risk assessment and identification of fall risk factors, skills required to do a fall risk assessment, and interventions for preventing falls. Nurses were also given a pre- and post-education knowledge test to assess their learning following these sessions.

Lack of resources	Reminders & Identification systems	Reminder methods included the mandatory fall risk assessment tool incorporated in nursing assessment notes, prompting nurses to perform fall risk assessment upon admission and at every change of shift. All nurses were also given a pocket card which detailed the summary of the recommendations in the CPG. Posters on the Fall Prevention CPG were posted in all the participating wards to remind health care providers to use the guidelines.
		Identification systems were used to alert staff to patients assessed as at risk of a fall. These systems included: 1) pink name cards above the bed; 2) pink stickers on clinical/nursing notes; and 3) pink identification bracelets on the high risk patient.

Lack of facilities	Improved facilities	Improved facilities such as night lights, bed alarm devices, and facilities to maintain the equipment, were available in all the participating wards.

Lack of motivation	Audit and feedback	Audit and feedback strategies were employed with aggregate audit data on incidence of falls and compliance to use of the fall risk assessment tool, being posted in the department tea room at monthly intervals. The data were presented as simple tables and text, with feedback highlighting good practice, areas requiring improvement, and suggestions on how to achieve the change. Incentives such as McDonalds' vouchers were given monthly to the staff of the ward with the lowest fall rate and highest compliance to fall risk assessment.

### Study Limitations

Reporting bias associated with the self-report method raises questions about the extent to which the responses accurately represent nurses' actual experiences of barriers to change. That is, nurses may have reported 'socially acceptable' responses when completing the questionnaire. The written survey could have excluded other responses or items that were perceived by nurses as barriers to change. Additionally, there was limited psychometric testing of the revised tool, given that six questions were removed and six questions were reworded to address the practice guideline. The re-worded questions were pilot tested for understandability and relevance in the Singapore context, but have not undergone extensive psychometric testing.

## Conclusion

In conclusion, a survey design was used to elicit nurses' perceptions regarding barriers to implementation of the Fall Prevention CPG in their practice setting. The greatest barriers to implementation of guidelines reported included: 1) lack of knowledge and education and motivation of staff; 2) lack of change champions availability of support staff; and 3) lack of access to resource facilities. The findings of this study informed the development of a multifaceted strategy, with tailored interventions designed to target the identified barriers to implementation of the Fall Prevention CPG, so as to promote guideline implementation and evidence-based nursing practice in Singapore.

## Competing interests

The authors declare that they have no competing interests.

## Authors' contributions

SSLK, EM, AMH, LJ study  design, SSLK, SD data analysis, SSLK, EM, AMH, LJ manuscript preparation,  SSLK literature review.

## Pre-publication history

The pre-publication history for this paper can be accessed here:


